# Students’ perception and scores in Paediatrics end-of-clerkship and final professional Objective Structured Clinical Examination (OSCE): A comparative study

**DOI:** 10.12669/pjms.37.2.3422

**Published:** 2021

**Authors:** Sabeen Abid Khan, Sahira Aaraj, Sidra Talat, Nismat Javed

**Affiliations:** 1 Sabeen Abid Khan, MBBS, FCPS. Assistant Professor, Department of Paediatrics, Shifa College of Medicine, Shifa Tameer-e-Millat University, Islamabad, Pakistan; 2 Sahira Aaraj, MBBS, FCPS. Assistant Professor, Department of Paediatrics, Shifa College of Medicine, Shifa Tameer-e-Millat University, Islamabad, Pakistan; 3 Sidra Talat, MBBS, FCPS. Registrar, Department of Paediatrics, Shifa College of Medicine, Shifa Tameer-e-Millat University, Islamabad, Pakistan; 4 Nismat Javed, Final year medical student, Shifa College of Medicine, Shifa Tameer-e-Millat University, Islamabad, Pakistan

**Keywords:** Students’ perception, End-of-clerkship, Final professional, OSCE

## Abstract

**Objectives:**

This study aims to understand students’ perception of the usefulness of end-of-clerkship (EOC) as compared to professional exam Objective Structured Clinical Examinations (OSCE) and to compare student performance in both examinations.

**Methods:**

We conducted a cross-sectional study of medical students who were studying in the final year at Shifa College of Medicine, Shifa Tameer-e-Millat University, and appeared in both end-of-clerkship and final professional exams. The study was conducted in October 2019. The data was collected through a self-constructed questionnaire. The scores of all participants were also recorded. The data obtained was analyzed on IBM’s statistical package for the social sciences (SPSS) version 23 (IBM, Armonk, NY). Descriptive statistics were used for qualitative variables. Mean and standard deviation was calculated for quantitative variables. Chi-square test was also applied to assess for significant differences.

**Results:**

Out of 115 participants, 57 (49.6%) were males and 58 (50.4%) were females. Most of the participants (75.7%) agreed that both OSCEs were well-structured. Participants found that both the exams give a good cross-section of paediatrics and allowed them to learn something new (p=0.00). 45% of participants perceived that the end-of-clerkship OSCE exam helped in orienting them for the final professional exam by identifying their weaknesses.

**Conclusion:**

Although the EOC OSCE served as a good preparatory exercise, it did not alleviate the stress levels. However, the results in both OSCEs were comparable and students were satisfied with the current weightage of marks distribution.

## INTRODUCTION

The assessment of a student’s clinical competence is of paramount importance, and there are several means of evaluating student performance in medical examinations. The Objective Structured Clinical Examination (OSCE) is an approach to assess students in aspects of clinical competence and is evaluated in a comprehensive, consistent and structured manner, with close attention to the objectivity of the process.^1^ This method has become a standard and universal format of assessment with good reliability and validity.^2^

The OSCE is superior to the oral clinical examination because it assesses a broad area of competency, resulting in better reliability and validity.^3^ The OSCE has demonstrated validity and reliability for assessing medical trainees’ clinical skills in multiple disciplines.^3^,^4^ Literature supports the use of OSCEs to evaluate clinical knowledge and competence, professional judgment, problem-solving skills, and interpersonal and communication skills.^5^-^7^ The combination of OSCE with standardized board examinations has the potential to become the gold standard for measuring physician competence.^8^ However, on the other hand conducting an OSCE needs a dedicated team, time for preparing the exam, logistic support, and financially more costly as compared to other means of assessment.

OSCE was introduced in 1975 and first described as an assessment format in Paediatrics (Child Health) in 1980.^9^,^10^ since its inception, the OSCE has been increasingly used to provide formative and summative assessment in various medical disciplines worldwide, including non-clinical disciplines.

At Shifa College of Medicine, OSCE is being used as an assessment tool since 2009. We use OSCE as part of assessment in both end of clerkship (continuous assessment) and final professional Bachelor of Medicine, Bachelor of Surgery (MBBS) exit exams in all clinical subjects. In Paediatrics, students appear in end-of-clerkship (EOC) OSCE after eight weeks of structured clinical clerkship. End-of-clerkship assessments contribute 40% marks to final professional assessment. The major difference between the end-of-clerkship and final professional exam is the presence of external examiners in the later exam. Apart from this, the format, number of stations, and a variety of cases, timing at each station are the same. A table of specification is followed in both OSCEs to increase its reliability.

Despite OSCEs being a good tool for assessment, studies have not been done as to how students perceive the significance of the End-of-Clerkship (EOC) exam in preparing them for the final professional exam. As a part of the learner-based teaching process, we aim to understand students’ perception on usefulness of end-of clerkship as compared to professional OSCEs, particularly concerning satisfaction about the weightage of both exams and to compare their performance in both examinations in an attempt to determine if stress levels are managed differently for both exams. The study will highlight areas of improvement and feedback from students to further improve the assessment process.

## METHODS

This is a cross-sectional analytical study. The study included 115 final year students who appeared in the final professional Paediatrics exam held by Shifa College of Medicine, Shifa Tameer-e-Millat University. The study was conducted in October 2019. Students had already taken their End of Clerkship Paediatric exam. The study was approved by the IRB of Shifa International Hospital (IRB#256-746-2019). Informed consent was taken from students. Data was collected on a structured questionnaire.

The questionnaire comprised of questions regarding the structure and attributes of both end of clerkship and final professional OSCE. It also consisted of statements such as fairness of examination, weightage of the end-of-clerkship OSCE, gender, ethnicity, attitude of examiners, adequacy of time. The participants were given a 5-point Likert scale varying from ‘Strongly Disagree’ to ‘Strongly Agree’ to assess the degree to which the aforementioned statements had a role in affecting the OSCE scores and structure as a whole.

Participants were also instructed to comment upon stress levels, curriculum, and usefulness of end-of-clerkship OSCE experience in helping and stimulating them for better performance in final professional OSCE. Marks obtained by students in both OSCEs were also compared.

The final professional OSCE exam continued for four days which was also the duration of the study. 117 students were divided into four groups, each group comprising of 29 or 30 students each. On each day, groups were further divided into subgroups of 12 to 13 students each, and OSCE was run in two circuits. Table of specifications is shown in Table-I.

**Table-I:** Table of specifications.

*Topics*	*Group D*	*Group C*	*Group B*	*Group A*
Long Case 1 (History taking)				
Long Case 2 (clinical examination)				
Long Case 3 (Discussion with examiner)				
Short case 1 Clinical examination				
Short case 2 Clinical examination				
Table Viva/demonstration of skill				
X-Ray interpretation				
Picture Spot Diagnosis				
Anthropometry				
Emergency management				
Data interpretation				
Neonatal Resuscitation				

There were a total of 12 stations in both OSCEs. There were eight interactive and four static stations. The duration (including transit time) for each station was five minutes. There were three observed stations for ‘Long Case’ and two observed stations for ‘Short Case’. The total score for both OSCEs was 100 points each. A similar format was followed for end-of-clerkship OSCE for each group that attended the Pediatric rotation. The table of specifications was the same.

At the end of the OSCE, a questionnaire was distributed to all the students. These questionnaires were filled out in the presence of a preceptor to ensure that all queries be addressed and responses are recorded appropriately. The data obtained was analyzed on IBM’s statistical package for the social sciences (SPSS) version 23 (IBM, Armonk, NY). Descriptive statistics were used to analyze and describe the data. Frequencies and percentages were calculated for qualitative variables like gender. Mean and standard deviation (SD) were calculated for quantitative variables like scores.

## RESULTS

One hundred fifteen (98.3%) students completed the questionnaire and were included as participants of the study. There were 57(49.6%) male participants and 58(50.4%) female participants. The students had a mean score of 66.03±10.18 in end-of-clerkship OSCE and 64.73±8.00 in final professional OSCE. The minimum score obtained was 31.60 in end-of-clerkship OSCE and 43.19 in final professional OSCE. The maximum score obtained was 84.16 in end-of-clerkship OSCE and 82.80 in final professional OSCE.

Most of the students (65.0% to 75.7%) commented positively about the structure of both end-of-clerkship and final professional OSCE. Participants found that both the exams gave a good cross-section of paediatrics and posed an opportunity to learn something new. A similar set of statements was used in the questionnaire for final professional OSCE. These results have been further explained in Table-II and III.

**Table-II:** Students’ perception of end-of-clerkship OSCE.

*Structure*	*Strongly disagree (N, %)*	*Disagree, (N, %)*	*Neutral, (N, %)*	*Agree, (N, %)*	*Strongly agree, (N, %)*
Exam was fair	2 (1.7%)	1 (0.9%)	24(20.9%)	52(45.2%)	36(31.3%)
Exam was stressful	5 (4.3%)	9 (7.8%)	27(23.5%)	47(40.9%)	27(23.5%)
Exam was well-structured	1(0.9%)	5 (4.3%)	18 (15.7%)	59(51.3%)	32(27.8%)
Adequate time available at each station	15(13.0%)	44(38.3%)	33(28.7%)	18(15.7%)	5(4.3%)
More weightage assessment is required towards final OSCE	7(6.1%)	21(18.3%)	48(41.7%)	26(22.6%)	13(11.3%)
Gender and ethnicity did not affect exam	3 (2.6%)	3 (2.6%)	12 (10.4%)	39 (33.9%)	58 (50.4%)
Examiners were polite and professional	1 (0.9%)	5 (4.3%)	11(9.6%)	40 (34.8%)	58(50.4%)
Exam was a good cross-section of paediatrics	2(1.7%)	5 (4.3%)	24 (20.9%)	55 (47.8%)	29(25.2%)
Attributes					
Preparation of exam took more time	6 (5.2%)	21 (18.3%)	30 (26.1%)	44 (38.3%)	14 (12.2%)
Helped me identifying strengths and weaknesses	0 (0.0%)	9 (7.8%)	28(24.3%)	63(54.8%)	15(13.0%)
Provided feedback	3(2.6%)	15(13%)	39(33.9%)	41(35.7%)	16(13.9%)
No role for final professional OSCE	29(25.2%)	35(30.4%)	28(24.3%)	15(13%)	8(7%)
Improved performance in final professional OSCE	2(1.7%)	10(8.7%)	35(30.4%)	52(45.2%)	16 (13.9%)
Stimulation for final professional OSCE by giving appropriate scores	3(2.6%)	8(7.0%)	27(23.5%)	59(51.3%)	18(15.7%)
Motivation to learn new concepts	1(0.9%)	7(6.1%)	38(33.0%)	52(45.2%)	17(14.8%)

**Table-III:** Students’ perception about final professional OSCE.

*Structure*	*Strongly disagree (N, %)*	*Disagree (N, %)*	*Neutral (N, %)*	*Agree (N, %)*	*Strongly agree (N, %)*
Exam was fair	1(0.9%)	3(2.6%)	21(18.3%)	65(56.5%)	25(21.7%)
Exam was stressful	0(0.0%)	9(7.8%)	18(15.7%)	43(37.4%)	45(39.1%)
Exam structure differed from EOC OSCE	8(7.0%)	45(39.1%)	28(24.3%)	23(20.0%)	11(9.6%)
More time required at stations	11(9.6%)	44(38.3%)	31(27.0%)	21(18.3%)	8(7.0%)
Gender and ethnicity did not affect exam	6(5.2%)	5(4.3%)	17(14.8%)	38(33.0%)	48(41.7%)
Examiners were strict	4(3.5%)	20(17.4%)	37(32.2%)	33(28.7%)	20(17.4%)
Exam was a good cross-section of paediatrics	2(1.7%)	8(7.0%)	33(18.7%)	53(46.1%)	19(16.5%)
Attributes					
Prior orientation helped in preparation	2(1.7%)	1(0.9%)	30(26.1%)	57(49.6%)	25(21.7%)
OSCE scores provide true measure of essential clinical skills	3(2.6%)	7(6.1%)	41(35.7%)	52(45.2%)	12(10.4%)
More intimidating more than end-of-clerkship OSCE	0(0.0%)	7(6.1%)	25(21.7%)	37(32.2%)	46(40.0%)
Commitment for optimal performance	4(3.5%)	10(8.7%)	30(26.1%)	43(37.4%)	28(24.3%)
Preparation of exam took a lot of time	2(1.7%)	16(13.9%)	33(28.7%)	46(40.0%)	18(15.7%)
Helped me identify my strengths and weaknesses	0 (0.0%)	4(3.5%)	34(29.6%)	58(50.4%)	19(16.5%)
Motivation to learn new concepts	1(0.9%)	7(6.1%)	30(26.1%)	46(40.0%)	31(27.0%)

We also compared the differences in percentage when similar statements were brought into consideration. Chi-square was used to test if these differences were significant. P-value less than 0.05 was considered significant. This is shown in Table-IV.

**Table-IV:** Comparison of attributes of both OSCEs.

*Attributes*	*End-of-Clerkship OSCE*	*Final professional OSCE*	*P-value*
Exam was stressful	74(64.3%)	88(76.5%)	0.00
Gender and ethnicity did not affect exam	97(84.3%)	86 (74.7%)	0.00
Examiners were stricter	17(14.8%)	53 (46.1%)	0.95
Examiners were polite	98(85.2%)	24(20.9%)	0.95
OSCE provided good cross-section of paediatrics	84(73.0%)	72(62.6%)	0.00
Final professional OSCE is more intimidating	74(64.4%)	83(72.2%)	0.00
Exam preparation took a lot of time	58(50.5%)	64(55.75%)	0.00
Helped me identify my strengths and weaknesses	78(67.8%)	77(66.9%)	0.00
Prior orientation helped in Final Professional OSCE	68(59.1%)	82(71.3%)	0.17
Motivation to learn new concepts	69(60.0%)	77(67.0%)	0.00
Similar to real life encounters	69(60.0%)	73(63.5%)	0.00
Exam was well structured	34(29.6%)	91(79.1%)	0.03

## DISCUSSION

OSCE is considered an excellent assessment tool for clinical skills.^11^ In our set up it’s part of our continuous assessment and final professional exit exam and accepted well by students. Students agreed that both OSCEs provided a good cross-section of Pediatrics cases (p=0.00) and provided real-life scenarios. Students agreed that both OSCEs were well structured (p=0.03). Gender and ethnicity did not affect the results (P=0.00). This is in contrast to other studies done internationally where students were neutral that OSCE served the purpose of motivation to learn new concepts.^12^ Regarding the reliability of scoring, students agreed that the results were reflective of their performance (p=0.00).

When a comparison of both OSCEs was done the study identified that 45% of participants perceived that end-of-clerkship Pediatrics OSCE helped them prepare better for their professional exams. 13 It served a good purpose in identifying their areas of weaknesses (p=0.00). This has been advised in other studies on the significance of OSCE in clinical exams that orientation to this exam format is important for students to perform better.^14^ However, the majority (53.0%) of the participants agreed that more weightage should be given to the final professional exam. This is attributed to the fact that 61.7% of the participants took the final professional exam more seriously than the end-of-clerkship exam. Furthermore, EOC assessment serves as a good orientation exercise to help them be more prepared in Final professional OSCE.^15^ Participants (76.5%) found the professional exam to be more intimidating and stressful (p=0.00) 16. Prior experience in terms of EOC OSCE did not seem to alleviate this stress. The same trends have been seen in other studies that anxiety and stress levels run high in Professional exam.^17^ In our study, the presence of external examiners can also be a factor contributing to their stress levels. 18 Students believed that examiners were stricter in the final professional exam (46.1%) as compared to the EOC exam (14.8%).^19^

Comparing the scores of both OSCEs the minimum marks obtained in FP OSCE were higher than EOC highlighting improved performance (31.6 versus 43.9).This study highlighted the fact that EOC OSCE is perceived by students as a good tool for orienting them to final professional OSCE. 20 It also helps them identify areas of improvement and work on their clinical skills as observed by improved minimum scores in Final professional OSCE. Students (59%) agreed that marks in internal assessment also served as a stimulus to perform better in professional exams. It focuses on customized learning for each individual. Students contended with the 40 % weightage of EOC exam results contributing to the final exam scores (67.8%).

### Limitation of the study

This study was performed in one discipline only and the weightage of continuous assessment can be different from other institutions. These examinations should be implemented in other institutes and not only medical schools so that an organized framework is brought to the learner’s mind when such situations arrive in the practical and professional lives of the learners.

## CONCLUSIONS

Although the EOC OSCE served as a good preparatory exercise, it did not alleviate the stress levels. However, the results in both OSCEs were comparable and students were satisfied with the current weightage of marks distribution.

### Authors’ Contributions:

**SAK:** Conception of study, data collection, accuracy and integrity of the work.

**SA:** Drafting of study, interpretation of results.

**ST:** Data collection & entry; analysis.

**NJ:** Final revision and drafting.

## References

[1] Bergus GR, Woodhead JC, Kreiter CD (2009). Trained lay observers can reliably assess medical students'communication skills. Med Educ.

[2] Majumder MAA, Kumar A, Krishnamurthy K, Ojeh N, Adams OP, Sa B (2019). An evaluative study of objective structured clinical examination (OSCE):students and examiners perspectives. Adv Med Educ Pract.

[3] Plakiotis C (2017). Objective Structured Clinical Examination (OSCE) in Psychiatry Education:A Review of Its Role in Competency-Based Assessment. Adv Exp Med Biol.

[4] Risse J, Busato T, Dufrost V, Perri M, Zuily S, Wahl D (2017). Development of an objective structured clinical examination (OSCE) for evaluating clinical competence in vascular medicine. J Med Vasc.

[5] Al-Naami MY, El-Tinay OF, Khairy GA, Mofti SS, Anjum MN (2011). Improvement of psychometric properties of the objective structured clinical examination when assessing problem solving skills of surgical clerkship. Saudi Med J.

[6] Graf J, Smolka R, Simoes E, Zipfel S, Junne F, Holderried F (2017). Communication skills of medical students during the OSCE:Gender-specific differences in a longitudinal trend study. BMC Med Educ.

[7] Naumann FL, Marshall S, Shulruf B, Jones PD (2016). Exploring examiner judgement of professional competence in rater based assessment. Adv Health Sci Educ Theory Pract.

[8] Eva KW, Bordage G, Campbell C, Galbraith R, Ginsburg S, Holmboe E (2016). Towards a program of assessment for health professionals:from training into practice. Adv Health Sci Educ Theory Pract.

[9] Harden RM, Stevenson M, Downie WW, Wilson GM (1975). Assessment of clinical competence using objective structured examination. Br Med J.

[10] Waterson T, Cater JI, Mitchell RG (1980). An objective undergraduate clinical examination in child health. Arch Dis Child.

[11] Harden RM, Caincross RG (1980). The assessment of practical skills:the Objective Structured Practical Examination (OSPE). Stud High Educ.

[12] Delavar MA, Salmalian H, Faramarzi M, Pasha H, Bakhtiari A, Nikpour M (2013). Using the objective structural clinical examinations in undergraduate midwifery students Med Life.

[13] Raheel H, Naeem N (2013). Assessing the objective structured clinical examination:Saudi undergraduate medical students 'perceptions of the tool. J Pak Med Assoc.

[14] Müller S, Settmacher U, Koch I, Dahmen U (2018). A pilot survey of student perceptions on the benefit of the OSCE and MCQ modalities. GMS J Med Educ.

[15] Gormley G (2011). Summative OSCEs in undergraduate medical education. Ulster Med J.

[16] Elfaki OA, Al-Humayed S (2016). Medical Students'Perception of OSCE at the Department of Internal Medicine College of Medicine King Khalid University, Abha, KSA. J Coll Physicians Surg Pak.

[17] Kim KJ (2016). Factors associated with medical student test anxiety in objective structured clinical examinations:A preliminary study. Int J Med Educ.

[18] Hashmat S, Hashmat M, Amanullah F, Aziz S (2008). Factors causing exam anxiety in medical students. J Pak Med Assoc.

[19] Khursheed I, Usman Y, Usman J (2007). Students'feedback of objectively structured clinical examination:A private medical college experience. J Pak Med Assoc.

[20] Siddiqui FG (2013). Final year MBBS students perception for observed structured clinical examination. J Coll Physicians Surg Pak.

